# A Manufacturing Protocol for Complete Dentures Using a Milling Cutting Method with an Individual Correction of the Prosthetic Plane

**DOI:** 10.3390/bioengineering13080878

**Published:** 2026-07-30

**Authors:** Wojciech Kondrat, Anna Stocka, Paula Łasica, Mutlu Özcan, Teresa Sierpińska

**Affiliations:** 1 Department of Prosthetic Dentistry, Medical University of Bialystok, Sklodowska-Curie Str. 24a, 15-276 Bialystok, Poland; wojciech.kondrat@umb.edu.pl (W.K.); anna.stocka@umb.edu.pl (A.S.); paula.lasica@umb.edu.pl (P.Ł.); 2 Clinic of Masticatory Disorders and Dental Biomaterials, Center for Dental Medicine, Plattenstrasse 11, CH-8032 Zurich, Switzerland; mutluozcan@hotmail.com

**Keywords:** complete dentures, CAD/CAM manufacturing, milling

## Abstract

Purpose: This study describes the manufacturing procedure for complete dentures using a milling cutting method with an individual correction of the prosthetic plane. Methods: Complete dentures were fabricated digitally using a hybrid protocol with individual assessment of the prosthetic plane. Results: Subjective and objective studies of evaluation of dentures were used. The sampling of functional impressions with the use of silicone masses of decreasing tension on dentures was used by a patient after the functional formation of their edges with Function type masses. The recording of occlusion on the pre-prepared prosthetic impressions using a silicone recorder was performed. Scanning of dentures and their occlusive relations by means of a laboratory scanner was performed in order to obtain virtual working models. A 3D face photograph of the dentures was taken using the Face Hunter device. Face Hunter offered an individualised adjustment of the prosthetic plane and a unique incorporation of the restoration in a given subject. New restorations in a computer programme were designed. A milling cutting of try-in test dentures was performed in order to make a control in the oral cavity. Control of the test dentures took place at the dentist’s office. The performance of new prosthetic restorations using a milling cutting method in the PMMA target material was evaluated in this study. Conclusions: The Face Hunter device offers an individualised incorporation of dentures in patients’ faces, which considerably improves the aesthetic aspect of the complete dentures.

## 1. Introduction

The prevalence of tooth loss within the Polish population is a cause for concern. About 60% of individuals aged 40 were found to have up to five teeth missing, while about 15% of them were found to have the lack of 6–10 teeth in both jaws. About 50% of individuals aged 60 lacked 11–16 maxillary teeth. In approximately 35% of cases, an equal number of mandibular teeth were also missing. The proportion of seniors aged over 75 who are edentulous accounts for 54.6% in Poland.

Prosthetic rehabilitation of edentulous patients is an enormous challenge even for qualified clinicians. Despite proper manufacture of complete dentures, they may restore mastication only to a low degree. This is usually related to a poor condition of the denture-bearing area that reduces the resistance to vertical dislodgement (retention) and impairs the mechanisms coordinating denture use [1]. Lack of retention and stability of the complete denture affects the neuromuscular activity that influences the masticatory system. It was found that new technologies, materials and tools enable digital fabrication of complete dentures and may significantly improve denture aesthetics and function [2,3,4,5,6,7,8,9,10,11,12,13,14,15,16,17,18,19,20,21,22,23,24,25,26]. The protocol applied for analogue technologies of denture manufacturing has been known for many years. One of its key points is to determine the prosthetic plane. Accurate determination of this plane is essential for both the proper function and aesthetics. During standard prosthetic procedures, the prosthetic plane is established based on average measurements. As a rule, it is determined in relation to the Camper’s plane or in relation to the Frankfurt plane. Unfortunately, the mentioned procedures do not allow for individualisation. In contrast, modern technologies allow for an accurate digital determination of this plane, and significantly influence their aesthetics and comfort in use. These recent technological advancements make it possible to eliminate errors that may occur using conventional methods, which rely on the clinician’s subjective assessment of the prosthetic plane.

Complete denture fabrication using digital workflows can be carried out in two ways:

1. As restorations made entirely using a digital protocol—currently, the quality of such a restoration cannot be accurately assessed due to numerous difficulties in scanning the denture foundation.
2. As restorations made in a hybrid mode, where functional impressions and centric occlusion records are obtained in the analogue protocol, and, subsequently, after the impression and the occlusion records have been scanned, the fabrication is continued using the digital protocol.

The aim of the study was to develop the protocol for the individualisation of the prosthetic plane during complete denture fabrication using a hybrid milling protocol.

## 2. Material and Methods

This study was performed on a group of 10 patients aged 52–79, average age 68.3 years, including six females (average age 67.3 years) and four males (average age 70 years).

### 2.1. Inclusion Criteria

• Complete edentulism.
• The use of complete dentures ≥ 5 years.
• The normal vertical dimension at centric occlusion in the complete dentures currently used by the patients.
• Age ≥ 50 years.
• Patients healthy in general.

### 2.2. Exclusion Criteria

- Prosthetic

• No history of removable denture use.
• Extremely difficult conditions of the denture-bearing area (cleft, the state post-mandibular resection, total alveolar resorption).
• Inflammatory lesions of the denture-bearing mucosa.
• Functional disorders of the masticatory system.
• Poor oral hygiene and the hygiene of previously used dentures.

b. Health-status-related exclusion criteria

• Disability or impaired ability to independently place and remove the denture and maintain its hygiene
• Diseases: neoplastic, type II diabetes, AIDS, mental disorders
• Smoking, alcoholism, drug abuse
• Use of specific drugs, e.g., steroids

The study was approved by the local bioethics committee No. APK.002.374.2021. All participants were informed of the study’s purpose and procedures. Written informed consent was obtained prior to participants’ inclusion.

**Figure 1:**
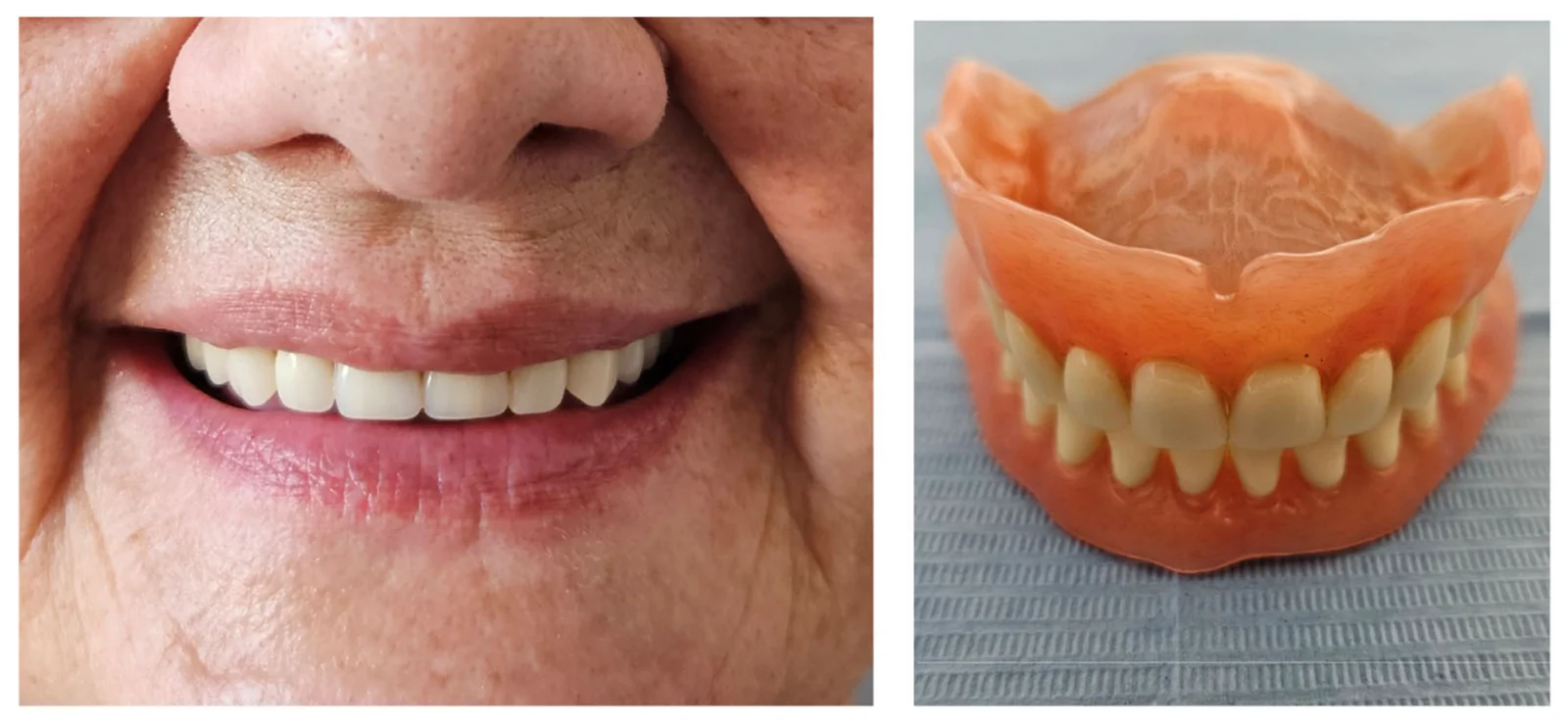
Patient looking in, smiling with currently used dentures manufactured in conventional protocol and dentures out of the mouth.

Patients were enrolled in the study based on medical history, clinical examination, assessment of denture quality, and evaluation of facial aesthetics (Figure 1).

**Figure 2:**
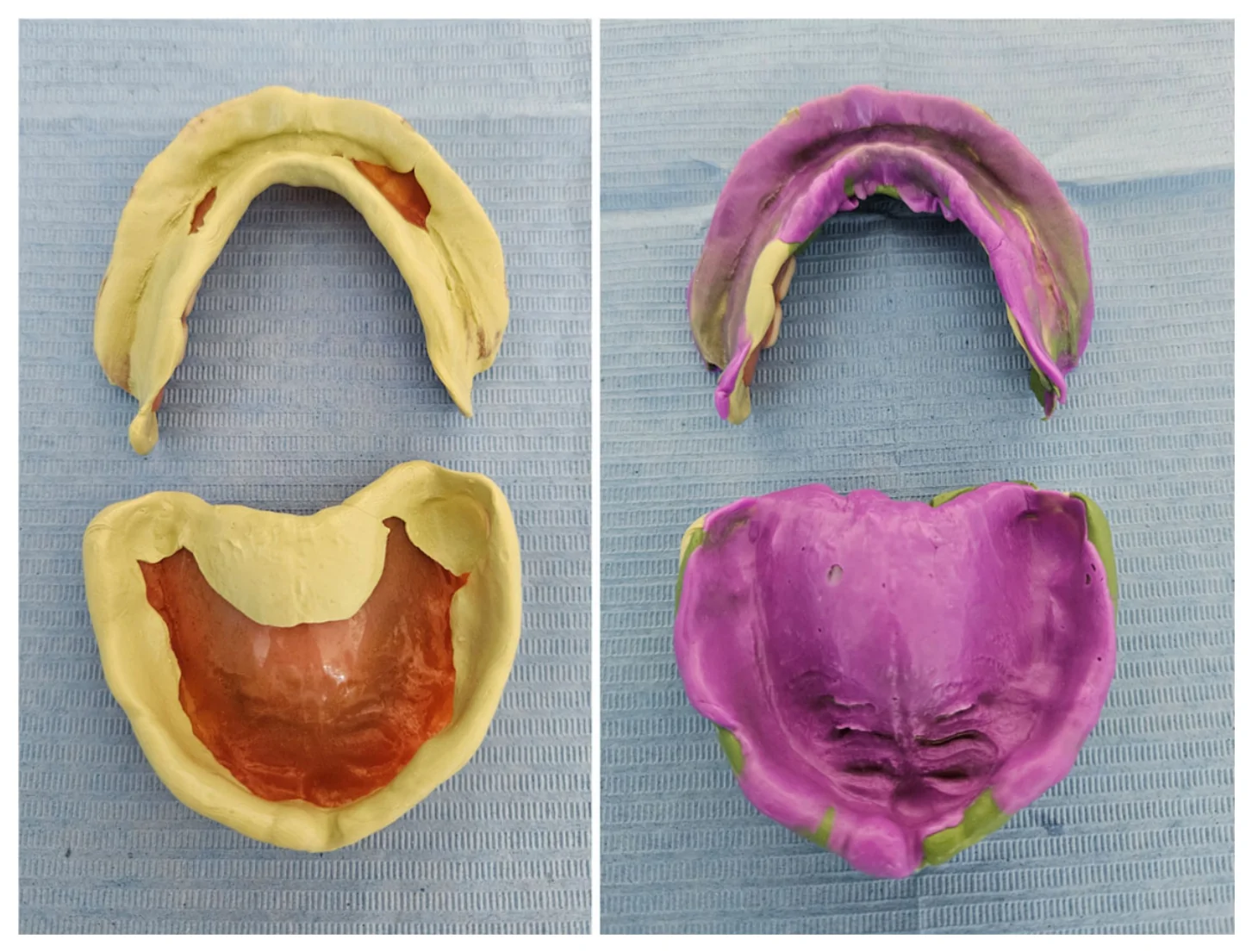
Functional impressions of an edentulous ridge obtained using a silicone material designed to apply progressively decreasing pressure on the patient’s denture after the functional formation of their borders using a Function-type material.

Next, a functional impression was taken using the patient’s existing denture. To this end, the denture was shaped using Function material (Bisico Function). Subsequently, impressions of the patient’s mandible and maxilla were simultaneously taken using Regular Body silicone material (Express, 3M) in the closed-mouth position, precisely modelling the patient’s denture foundation (Figure 2).

**Figure 3:**
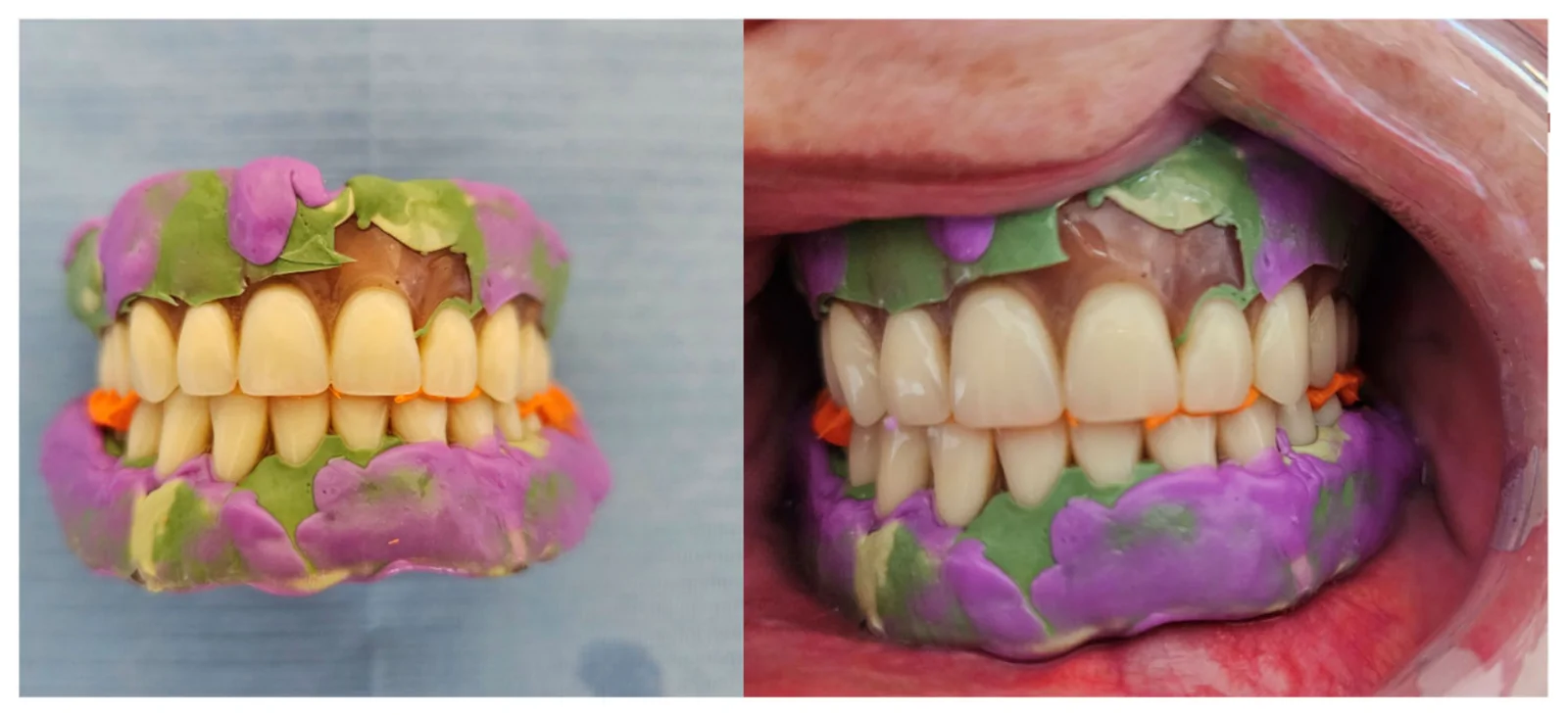
Occlusion recorded on the pre-prepared prosthetic impressions using a silicone recorder.

**Figure 4:**
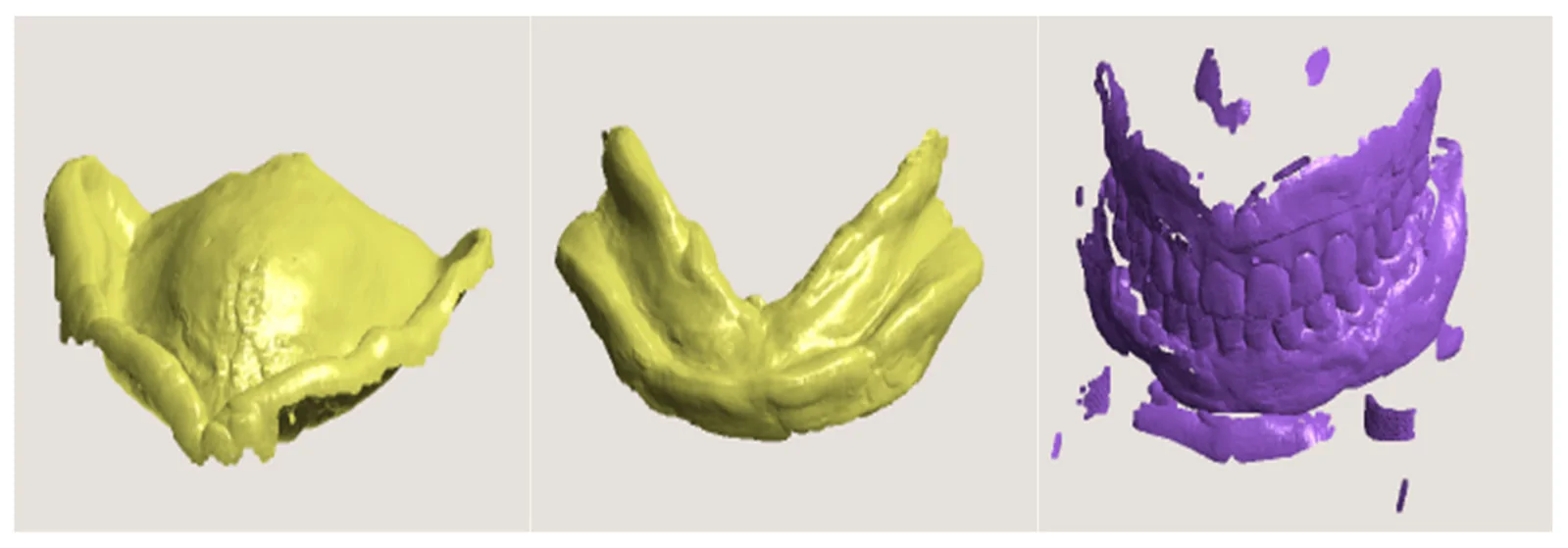
Scanning of impressions made on the old dentures and occlusive relations by means of a laboratory scanner in order to obtain virtual working models.

Once the functional impression was taken, when perforating the denture base, the procedure was repeated with the additional use of Light Body silicone material (Light Body, Express, 3M). The impressions properly made on the patient’s existing denture demonstrated good retention and stability. Occlusion was recorded on the patient’s denture relined with the silicone recorder (Figure 3). Extraoral imaging of the dentures was then performed using a laboratory scanner (Trios 5, 3Shape) to obtain virtual working models (Figure 4).

**Figure 5:**
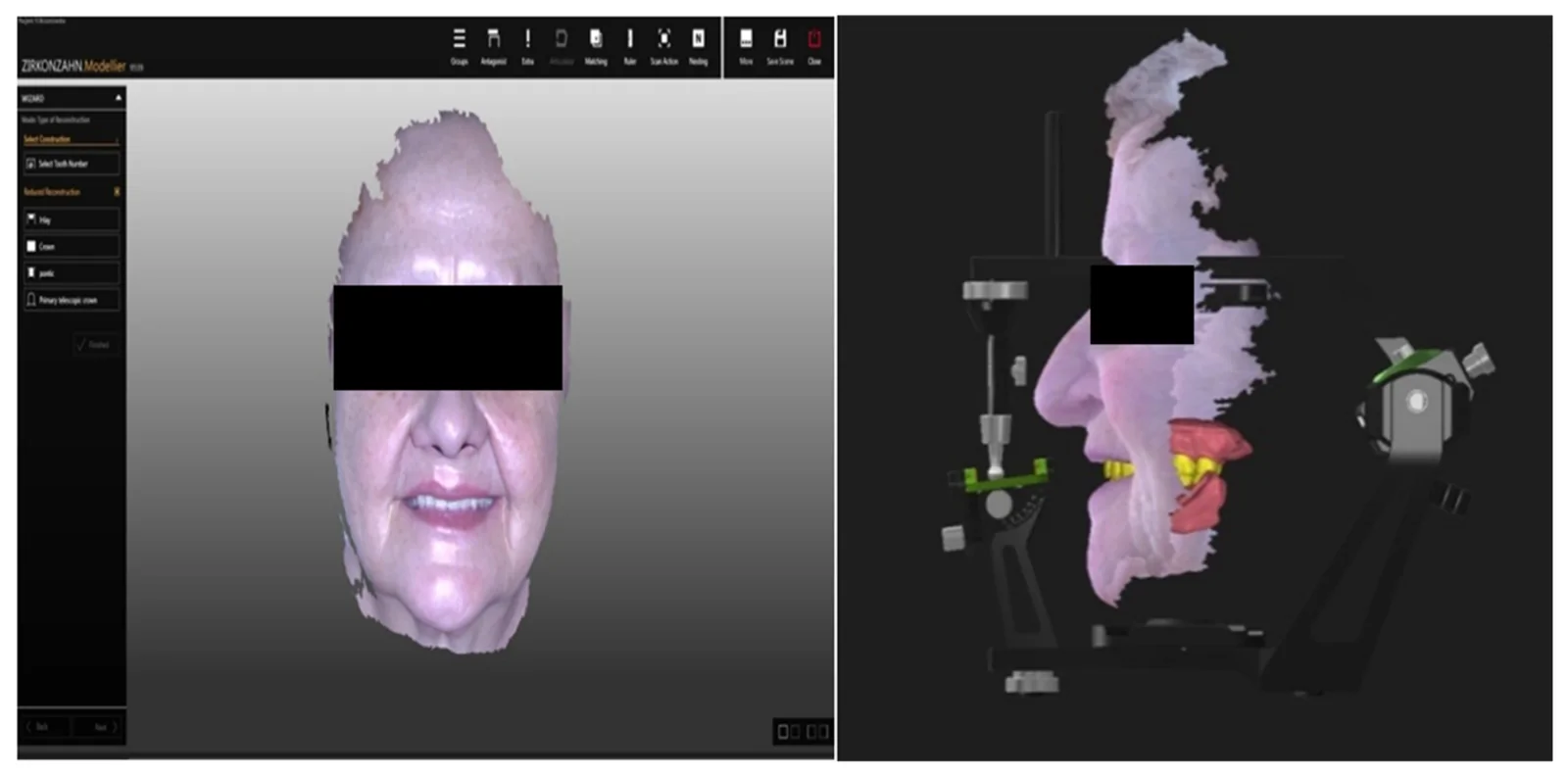
Taking a 3D facial image of the patient wearing dentures using a Face Hunter device. Face Hunter offers an individualised adjustment of the prosthetic plane and a unique incorporation of the restoration in the given subject.

The extraoral imaging was performed in the following way: dentures relined with silicone impressions were placed on a specially designed table inside the scanner’s chamber. The scanning mode of the given impression was selected. The selection activates the programme, which automatically converts the scan into a negative. To this end, the area to be scanned must be selected and the light intensity adjusted accordingly. Scanning should be continued until the desired image quality is achieved. This enables the creation of a model of the scan of the denture-bearing area. The impressions made using the patients’ dentures were scanned separately to obtain models of the maxilla and mandible. ‘Scan and Match’ is an additional function, which enables the scanning of an object divided into two parts, which are later assembled into a single unit. This function allowed us to scan the patient’s previously worn dentures (maxillary and mandibular) separately, which was followed by a combined scan of this patient’s dentures positioned and fitted together. After scanning, the patient’s dentures were returned to them. In the next stage, 3D facial images were made using the Face Hunter device (3D dental scanner Zirkonzahn) (Figure 5). The patient wore dentures during the scanning process. Face Hunter offers the possibility of individualised adjustment of the prosthetic plane. As a result, it provides the opportunity to introduce a prosthetic restoration, individually obtained, into the patient’s face. For this purpose, in our study, the patient was positioned sitting on a chair with their right hand extended forward to ensure the projector of Face Hunter was placed at a consistent distance. Next, the patient was asked to look into a mirror and to adjust the chair so that they could look straight into their own eyes in the mirror. The process of facial imaging was then started, which involved adjusting the light intensity in the program to achieve the best possible quality of the 3D image.

Images required for denture fabrication:

- Images en face and of the left and right profiles—these three images are combined to make a single block, creating an image of the patient’s whole face (ensure that the patient’s alae of the nose and ear lobes are fully visible in the images, as the Ala–Tragus plane is determined by referencing these images).
- An image taken while smiling.
- An image with the mouth open.

For recording purposes, the patient was given a bite-type silicone material on a specially designed “transfer fork” equipped with reference points detectable by the scanner. Subsequent images were taken in this arrangement.

**Figure 6:**
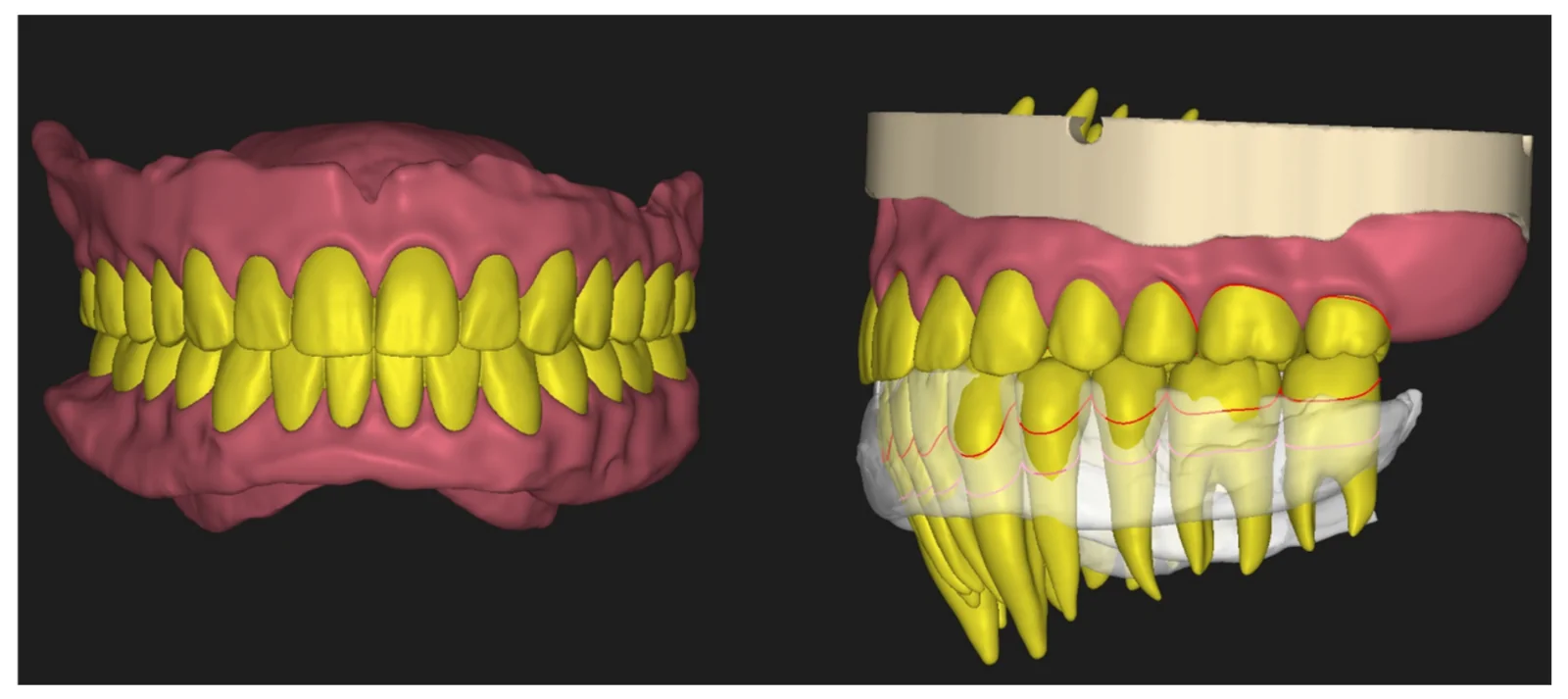
Designing new restorations in a computer programme.

After scanning, the Archive module was started, during which the type of restoration and the patient’s data were registered. The teeth in the upper and lower arch were marked. Next, the Model Maker software module was selected in the dialogue box. Once the module was opened, the previously scanned impressions of the maxillary and mandibular arches had to be downloaded to the device, and any redundant images trimmed and removed using the available tools. Subsequently, the software generated working models onto which the prosthetic restoration would be designed. The resulting models were saved to the designated folder. The next stage involved layering the “transfer fork” scan on the scans of the face obtained using Face Hunter. Achieving this was possible with scanner software. The first action required to achieve this was the import of the patient’s previously scanned upper denture, followed by scanning the “transfer fork” together with the patient’s teeth impressed in silicone. After scanning, with the use of the reference points, the software automatically aligned the resulting scan with the 3D scan of the patient’s face (this was the scan obtained using the same “transfer fork” yet derived from Face Hunter). Subsequently, based on the occlusion record, the denture scan was aligned with the scan obtained with the “transfer fork”. This made it possible to obtain the exact location of the patient’s maxilla. The next stage involved marking the following: the palatine raphe line, the position of the first molars, and the Ala–Tragus plane (for the right and left sides of the patient). The aforementioned steps were carried out within the scanner software. Once all reference points had been marked, the entire design was saved, and the software simultaneously returned to Archive. The subsequent step in the process entailed designing a complete denture using the Modifier software (modifier 8915) (Figure 6). After opening the software and accessing the working box, the patient’s facial image was displayed alongside a correctly positioned upper denture, digitally suspended in space. The following step involved importing the previously created working models alongside the lower denture scan and the occlusion scan. All the downloaded scans were then loosely positioned in the digital workspace. The upper model had to be aligned with the upper denture scan using the designated reference points. When the model fitted correctly into its designated position, the occlusion scan was aligned with the upper model, then the lower denture scan, and finally the scan of the lower model. This ultimately resulted in the occlusion determined on the basis of the patient’s previously worn dentures and the complete dataset necessary for fabricating new ones. In the next stage, tooth forms were selected, and the procedure of setting them in dental arches began. The software enables more precise control in tooth arrangement than conventional tooth-setting techniques. During the designing process, the alveolar process was visible, allowing the teeth to be arranged directly on it. Consequently, the inclination of individual teeth or groups of teeth could be controlled, along with their shapes and sizes. Denture scans and 3D Face Hunter-derived images facilitate the entire process. For instance, when a scan taken during smiling is illuminated, it is possible to accurately determine the appropriate vertical level at which the cervical margins of the teeth should be positioned. This also helps determine the appropriate midline position or allows for a comparison of the old denture with the new tooth arrangement to identify any further corrections needed. Illumination and rotation of the 3D structure of the patient’s face enables visualisation of the tooth arrangement within the entire oral cavity. Additionally, this enables alignment of the tooth arrangement plane with the Ala–Tragus line.

Once the teeth had been arranged according to the established principles of complete denture fabrication, the process of blocking the working models began, followed by delineation of the denture base coverage area. On the model prepared in this way, a denture base was subsequently generated, which could be freely modelled using the available software tools. The subsequent stage involved creating sockets for the teeth in the denture base and saving all constructions. The teeth were formed in three blocks outlining the coverage area of the upper denture: 13–23, 14–17, 24–27, and 33–43, 34–37, 44–47 of the lower denture (teeth and denture base were milled separately). The files prepared in this way were queued for milling and subsequently arranged within material blocks to try-in, using the Nesting software (Nesting 9095). Once the CNC path had been calculated and validated, all the constructions were milled. Subsequently, the finished denture bases, together with the teeth, were milled from the material blocks and subsequently joined manually. Once all the components had been joined, the teeth had to be bonded into the sockets of the denture base. The try-in dentures were taken to the surgery, where the tooth arrangement, occlusion, and tooth shade selection were assessed.

**Figure 7:**
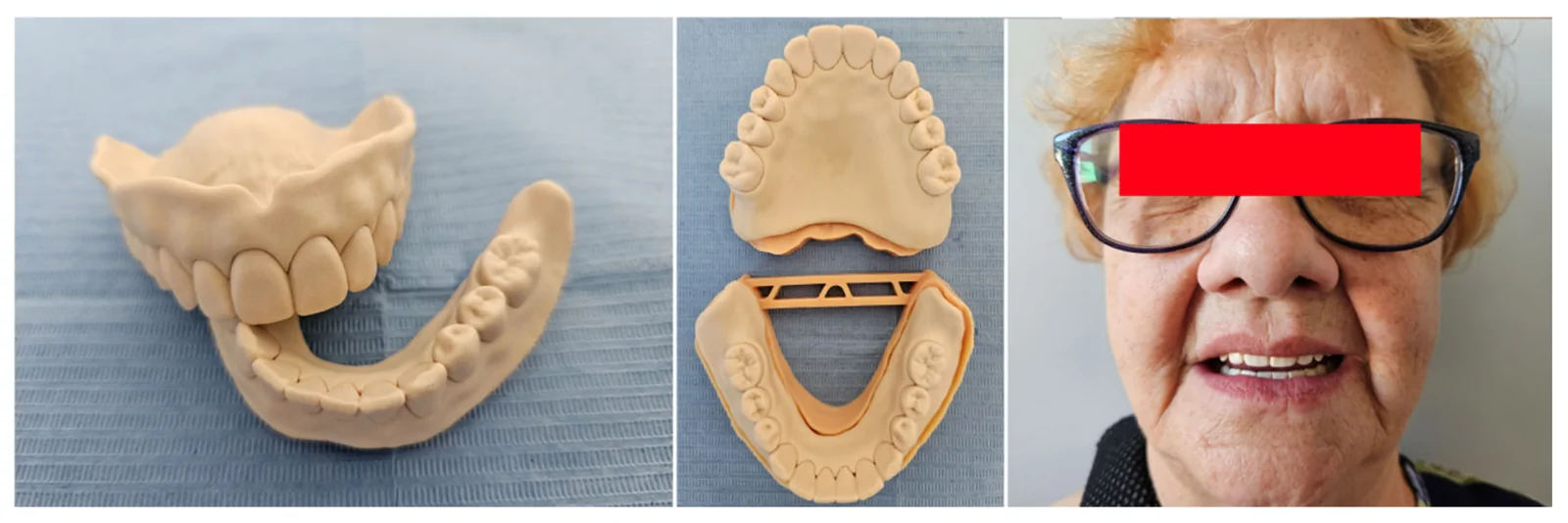
Milling of try-in dentures to perform a check in the oral cavity. Examination of the try-in dentures at the dental office.

**Figure 8:**
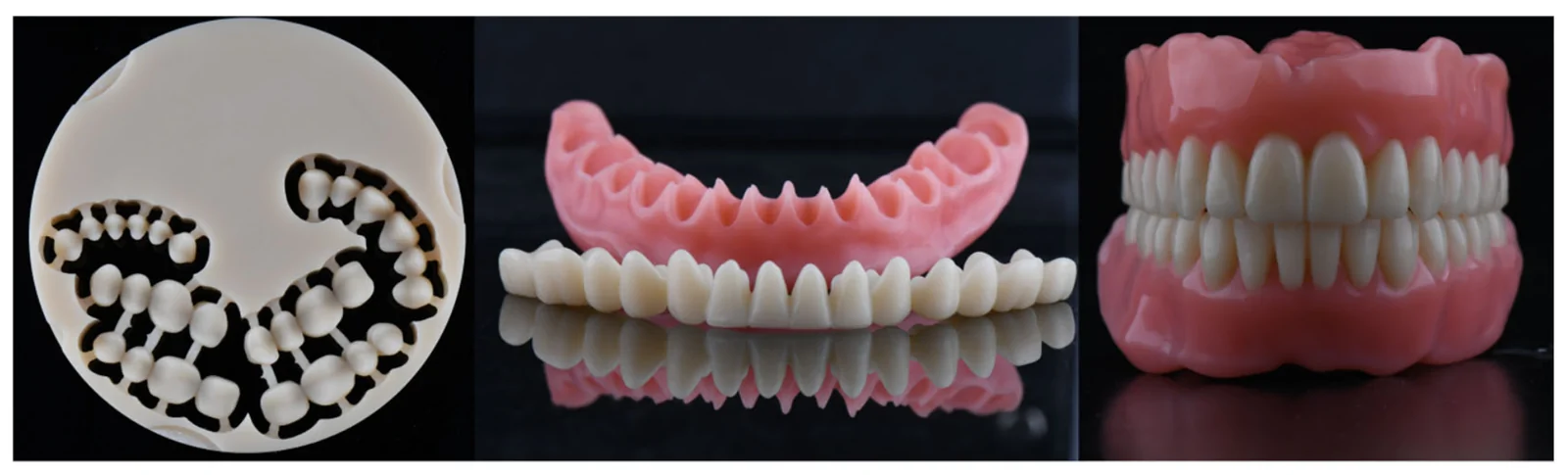
The performance of new prosthetic restorations using a milling method in the PMMA target material.

After the denture try-in at the office (Figure 7), the dentures that required no further adjustments were milled again (using the same files), but this time using the target material. Practically, no sufficient correction was required at the try-in stage. Only some small aesthetic discrepancies, such as angulation of incisors, were corrected. On the other hand, the corrected dentures, or those in which, e.g., teeth needed to be rearranged, were taken to the laboratory for scanning. This process involved starting the software and importing the trial denture scans, which enabled further modifications, including rearranging the teeth and realigning them with the fitting image. After readjusting the software and saving new versions, milling—the final stage of denture fabrication—could begin. Just like during “try-in milling”, the constructions needed to be placed in blocks and milling paths needed to be recalculated. The next step entailed milling the constructions, aligning them, and placing the teeth in the denture base sockets (Figure 8).

**Figure 9:**
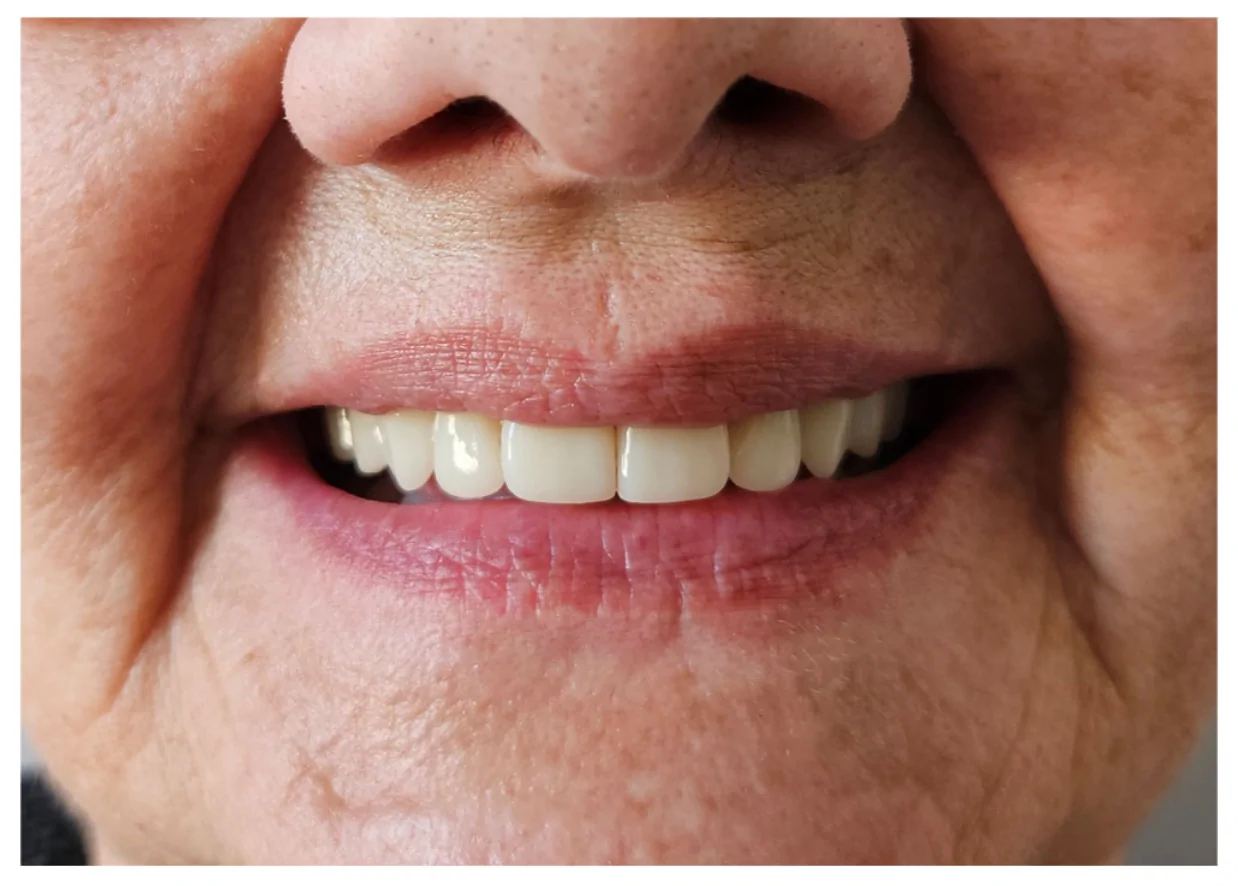
Denture fitting appointment at the dentist’s office.

Polibond (Gais, Zirkonzahn, Italy) was used to fix teeth to the denture base. This bonding agent partially dissolves the material, ensuring a sealed and durable bond of the whole construction. Immediate dentures were fabricated using milling, grinding stones, and rubber wheels, followed by final polishing. Dentures prepared following this procedure were delivered to the dental office for final fitting and handover to the patient (Figure 9).

## 3. Discussion

The recent development of digital technologies, including computer-aided design/computer-aided manufacturing (CAD/CAM) and 3D printing, has revolutionised the design and fabrication of complete dentures. They offer greater precision, faster processing times, and comfort for both patients and dentists, and the changes have been reported in detail in the literature [3,4,5,6,7,8,9,10,11,12,13,14,15,16,17,18,19,20,21,22,23,24,25,26]. In the context of digital prosthetics, the determination of the individual prosthetic plane is a key stage in the fabrication of complete dentures. The prosthetic plane enables the correct positioning of teeth in a denture. This makes the accurate determination of the plane essential for patient comfort. In the traditional method, the prosthetic plane used to be determined by manual procedures based on occlusion patterns, which sometimes required multiple adjustments within the oral cavity. In digital prosthetics, the aforementioned process has been considerably simplified owing to Face Hunter. The device enables the design of any individual prosthetic plane in a computer program. Furthermore, digital systems also allow analysis and optimisation of the prosthetic plane for aesthetics, which is particularly crucial in cases where a pleasing aesthetic effect is desired. The novelty of the protocol presented is to propose how to adopt a facial scanner for better assessment of the prosthetic plane when manufacturing complete dentures.

It is worth noting that determining the prosthetic plane individually using digital technology improves the quality of dentures, reducing the number of follow-up appointments and fabrication time. A reduced number of trial-and-error iterations during denture manufacturing represents savings for both the patient and prosthetic laboratory. Moreover, precise technologies facilitate improved denture adaptation to the oral soft tissues, which translates into greater comfort in use and enhanced functionality. Many authors [5,9,10,11,13] report greater satisfaction of their patients with the aesthetics of digital dentures. This is due to the fact that digital technologies enable greater individualisation of the denture in terms of the shade, shape and size of teeth, contributing to a more pleasant aesthetic effect. Dentures fabricated using the CAD/CAM method were also more accepted owing to a more comfortable fitting [5,7,8,21,25]. In contrast, despite proper fabrication, traditional dentures did not achieve such a high level of aesthetics and fitting as digital ones. Hsu et al. [24] report greater patient adaptation to dentures fabricated using digital milling. Other authors do not observe significant differences in their studies between the laboratory denture fabrication using the CAD/CAM method and the traditional one. According to Abdelnabi and Swelem [22], complete denture fabrication using 3D technology seems to be a promising avenue regarding denture fitting and aesthetics. However, clinical studies suggest that, for optimised functionality, some patients may still require denture adjustments. The authors of these studies also identified positive results in terms of comfort and masticatory function. Patients using 3D-printed dentures reported a better fit and fewer problems than dentures fabricated using the analogue method. In their investigation of masticatory force and performance, Ragheb and Ibrahim [23] pointed out that dentures fabricated using a digital protocol are more efficient than traditional ones. In particular, patients using digitally fabricated dentures reported greater comfort when eating hard foods. This suggests that the precision of fit provided by digitally fabricated dentures may enhance masticatory function. Similarly, the study of Mubaraki et al. [3], in which digital and analog dentures are compared in terms of fit, masticatory function, and general comfort, suggests that dentures made digitally offer a better fit and lower risk of maintenance issues, which may be especially important in the case of patients with anatomic issues. In their study, Steinmassl et al. [26] obtained similar results. The authors identified a better fit of dentures fabricated using the milling technique. Despite the many benefits offered by digital technologies, traditionally fabricated dentures are still widely used in prosthodontics. One of the key issues related to CAD/CAM technology in dental surgeries is the initial investment cost for the appropriate software and equipment. Furthermore, not every office has adequate infrastructure necessary for complete digital fabrication, which may significantly limit certain dentists, especially those in smaller practices. Although digital technologies in the fabrication of dentures offer many advantages, they also present certain challenges. For instance, the studies of Kattadiyil et al. [4,9,20] highlight concerns about the quality of materials used in CAD/CAM technology, including their durability over long-term use. Certain materials used in the fabrication of digital dentures may have inferior mechanical properties compared to those used in fabricating dentures by traditional methods. Biocompatibility and mechanical durability also present significant challenges, especially during long-term use [7].

The technology presented in this study is relatively new; therefore, it is necessary to extend the observation period and include a larger group of patients to confirm the quality of both the material and the technology.

## 4. Conclusions

Manufacturing complete dentures using a digital protocol with individualised adjustment of the prosthetic plane provides a broad basis for the fabrication of complete dentures characterised by high aesthetics and performance, simultaneously enabling even greater personalisation and reduced treatment time.

## Data Availability

All the data are available in the Department of Prosthetic Dentistry, Medical University of Bialystok, Poland.
